# Constraints on the jumping and prey-capture abilities of ant-mimicking spiders (Salticidae, Salticinae, *Myrmarachne*)

**DOI:** 10.1038/s41598-020-75010-y

**Published:** 2020-10-26

**Authors:** Yoshiaki Hashimoto, Tomoji Endo, Takeshi Yamasaki, Fujio Hyodo, Takao Itioka

**Affiliations:** 1 University of Hyogo/Museum of Nature and Human Activities, 6 Yayoigaoka, Sanda, Hyogo 669-1546 Japan; 2 School of Human Sciences, Kobe College, Okadayama 4-1, Nishinomiya, Hyogo 662-8505 Japan; 3 Research Core for Interdisciplinary Sciences, Okayama University, 3-1-1, Tsushimanaka, Okayama, 700-8530 Japan; 4 Graduate School of Human and Environmental Studies, Kyoto University, Yoshida-nihonmatsu, Kyoto, 606-8501 Japan

**Keywords:** Evolution, Mimicry, Zoology, Animal behaviour

## Abstract

Accurate morphological ant mimicry by *Myrmarachne* jumping spiders confers strong protective benefits against predators. However, it has been hypothesized that the slender and constricted ant-like appearance imposes costs on the hunting ability because their jumping power to capture prey is obtained from hydraulic pressure in their bodies. This hypothesis remains to be sufficiently investigated. We compared the jumping and prey-capture abilities of seven *Myrmarachne* species and non-myrmecomorphic salticids collected from tropical forests in Malaysian Borneo and northeastern Thailand. We found that the mimics had significantly reduced abilities compared with the non-mimics. The analysis using geometric morphometric techniques revealed that the reduced abilities were strongly associated with the morphological traits for ant mimicry and relatively lower abilities were found in *Myrmarachne* species with a more narrowed form. These results support the hypothesis that the jumping ability to capture prey is constrained by the morphological mimicry and provide a new insight into understanding the evolutionary costs of accurate mimicry.

## Introduction

**Figure 1:**
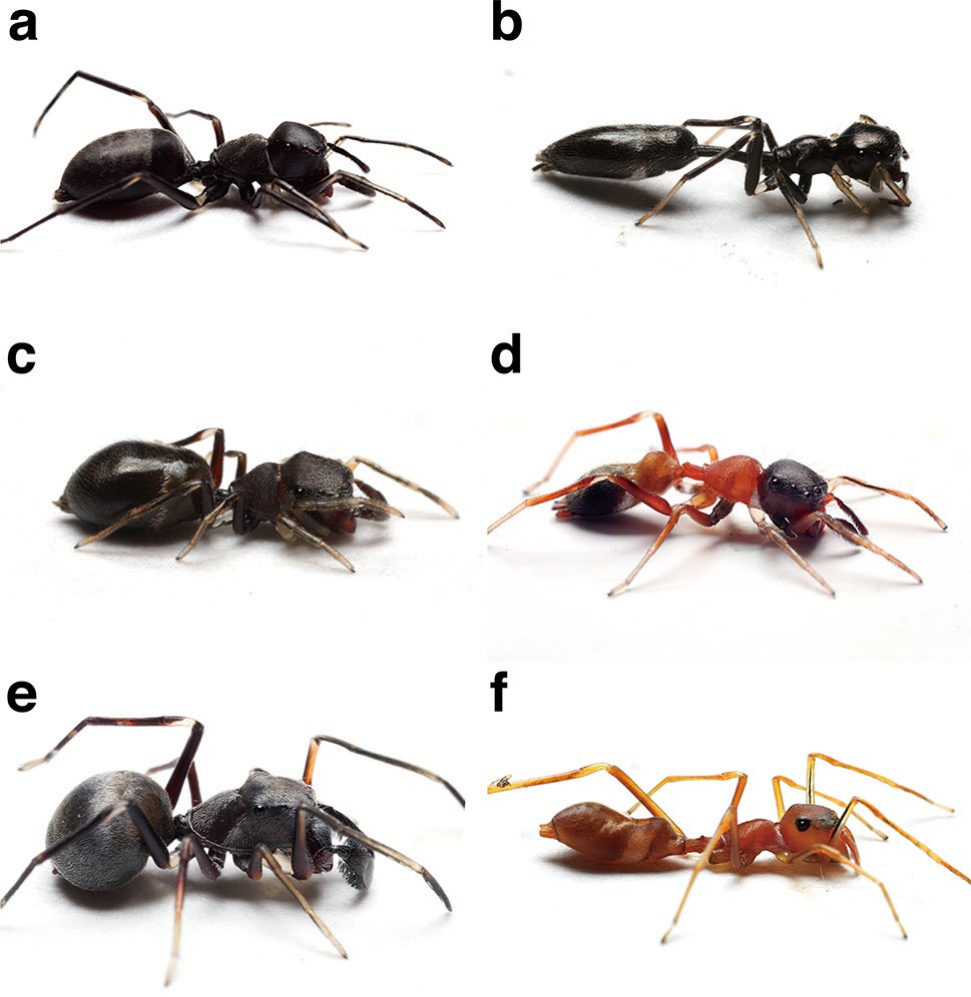
Studied species of *Myrmarachne* ant-mimetic jumping spiders, except *M. malayana*. (**a**) *Myrmarachne acromegalis*, (**b**) *M. cornuta*, (**c**) *M. hashimotoi*, (**d**) *M. melanocephala*, (**e**) *M. maxillosa*, and (**f**) *M. plataleoides*. (Photo credit: Hashimoto Y.).

Morphological ant mimicry is widespread in spiders^1^. Ants possess species-specific combinations of strong defensive traits, including powerful mouthparts and stings, chemical defenses, general aggressiveness, and nest-mate-recruiting ability^2^. Thus, morphological ant mimicry has been considered to be a good example of the Batesian mimicry^1^. Some of the most striking examples of morphological ant mimicry are found in the *Myrmarachne*, the largest genus of jumping spiders (Salticidae) with more than 200 species^3–5^. *Myrmarachne* has two body regions, a cephalothorax and an abdomen, but a constriction in the cephalothorax makes this portion resemble the separate head and thorax of an ant. The lengthened pedicel, in combination with a slender abdomen with a constriction in the anterior part, simulates the petiole and postpetiole segments of an ant abdomen and also enhances the spiders’ resemblance to that of ants. Additionally, most *Myrmarachne* species bear a striking resemblance to a particular species or genus of an ant^6^ (Fig. 1). For example, *Myrmarachne cornuta* is an accurate mimic of ants belonging to the genus *Tetraponera*, which have an extremely slender and elongated body shapes^7^. In contrast, *Myrmarachne maxillosa* has a more oval and stocky-shaped body, matching the appearance of the ants belonging to the genus *Polyrhachis*^8^. *Myrmarachne plataleoides* is a well-known accurate morphological mimic of the weaver ant *Oecophylla smaragdina* and has a short and convex cephalothorax and abdomen similar to the body form of its ant model^9^. The accurate ant-like appearances of *Myrmarachne* spiders confer protection against predation by visually oriented predators^10^, such as mantids^11,12^, and other spiders^13^.

Spiders are distantly related to insects and they have a completely different body plan. For example, unlike insects, spiders lack extensor muscles in major leg joints and instead use hydraulic pressure generated in their bodies to extend their legs for jumping^14^. To drive the hydraulic force for leg extension, spiders pump hemolymph fluid from the abdomen into the cephalothorax through the pedicel, and then increase the hemolymph pressure via a cephalothorax compression. In this hydraulic mechanism, sufficient body volume and hemolymph flow are required to produce a high fluid pressure^15,16^. Therefore, the slender and constricted ant-like appearances of *Myrmarachne* spiders are expected to constrain their jumping abilities. Indeed, earlier observations have suggested that, despite belonging to a jumping spider family, some species of *Myrmarachne* spiders were observed to be unable to jump and were only able to hunt prey in a close range^17^. However, such observations were based on a relatively few species and largely qualitative analyses. Furthermore, because each *Myrmarachne* species morphologically mimics the characteristics of a particular species or genus of an ant, the variations in the jumping abilities are expected to be associated with the variations in their ant-mimetic morphological traits. However, complex variation associated with body shape is a difficult type of variation to quantify^18^. Consequently, the relationship between the body shape and jumping ability in *Myrmarachne* spiders had not been explicitly tested before. In this study, we used geometric morphometric techniques (an approach that provides a mathematical description of biological forms) to quantify the body shapes of *Myrmarachne* spiders and test the hypothesis that a reduction in the jumping proficiency is associated with the shape adaptations related to ant mimicry. Since high species richness and morphological diversity of *Myrmarachne* spiders are found in tropical regions, particularly in Southeast Asia, where numerous ant species exist^3,18^, we collected *Myrmarachne* species with different body shapes and non-myrmecomorphic jumping spiders from three tropical forests in Southeast Asia. We then measured the jumping distances to capture prey and prey-capture success rates of seven *Myrmarachne* species and quantitatively compared the results with those of non-myrmecomorphic jumping spiders using geometric morphometrics. This study, for the first time, provides quantitative evidence to show that the accurate resemblance of *Myrmarachne* spiders to ants is likely to constrain their jumping ability for catching prey. In addition, our findings provide novel insights to further our understanding of the evolution and maintenance of accurate mimicry in ant-mimetic jumping spiders.

## Results

### Body shape comparisons

**Figure 2:**
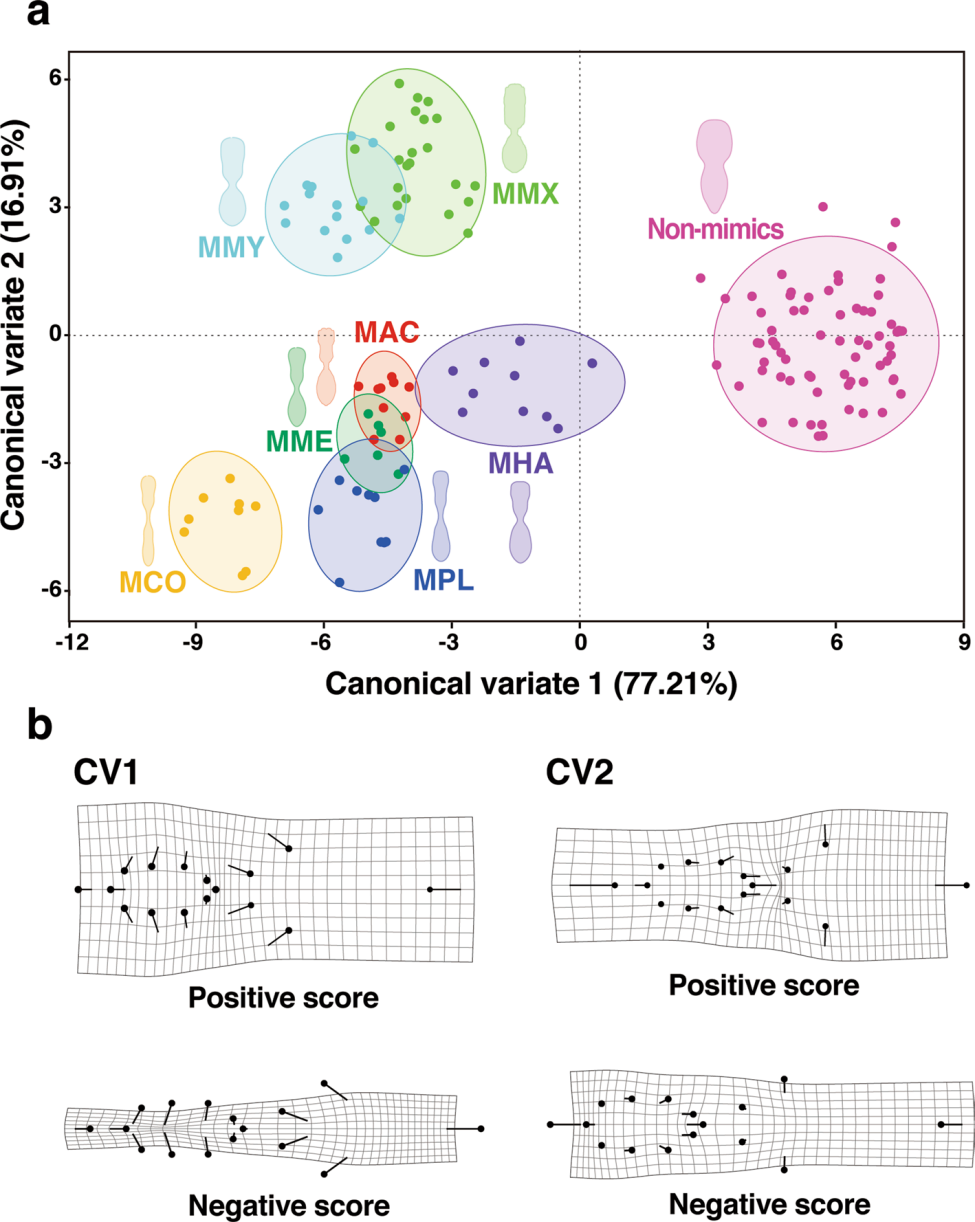
Differences in the body shape among *Myrmarachne* and non-myrmecomorphic jumping spiders. (**a**) Canonical variate analysis of seven *Myrmarachne* species and non-myrmecomorphic jumping spiders based on 16 landmarks of the body shape. Scatter plot of the first two canonical variate axes with 90% confidence ellipses (Non-mimics: Non-myrmecomorphic jumping spiders, MAC: *M. acromegalis*, MCO: *M. cornuta*, MHA: *M. hashimotoi*, MME: *M. melanocephala*, MMX: *M. maxillosa*, MMY: *M. malayana*, MPL: *M. plataleoides*). (**b**) Transformation grids illustrate the shape changes associated with the first two canonical variates in positive and negative directions with scaling factors of -9 to 9 for CV1 and -6 to 6 for CV2.

**Figure 3:**
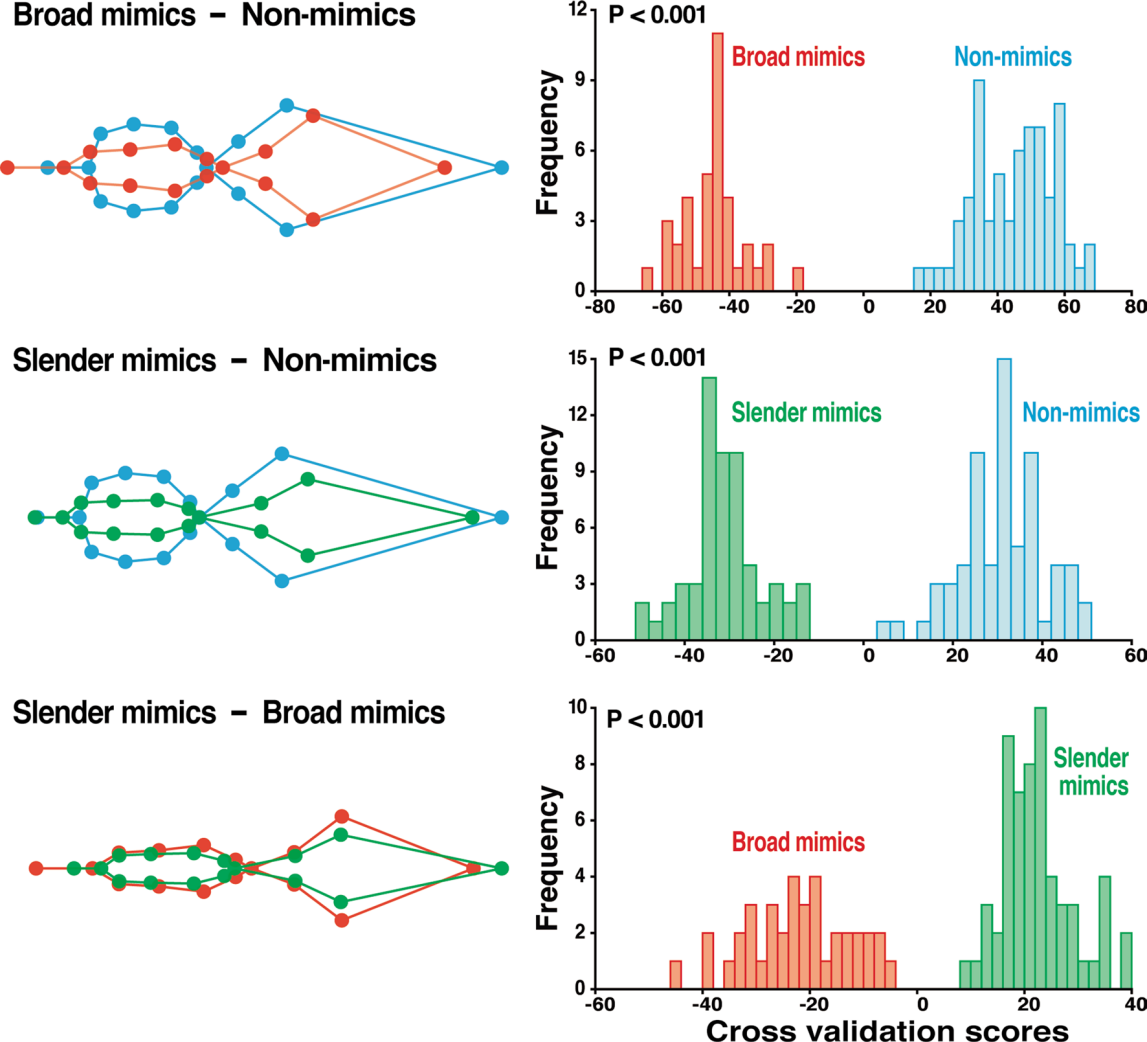
Discriminant function analysis for pairwise competitions among non-myrmecomorphic spiders (Non-mimics) and the two groups of *Myrmarachne* species (Broad mimics and Slender mimics). The mean body-shape differences of each group have been demonstrated with wireframe outlines (left). Cross validation scores of body-shape variables of each group have been shown as histograms (right). Permutation test with the T-square statistic for the Mahalanobis distance *P* < 0.001 for all comparisons. (Non-mimics: Non-myrmecomorphic jumping spiders; Broad mimics: *M. maxillosa* and *M. malayana*; Slender mimics: *M. acromegalis, M. cornuta, M. hashimotoi, M. melanocephala*, and *M. plataleoides*).

Canonical variate analysis (CVA) of the body shape of the seven *Myrmarachne* species (*M*. *acromegalis*, *M*. *cornuta*, *M*. *hashimotoi*, *M*. *malayana*, *M*. *maxillosa*, *M*. *melanocephala*, and *M*. *plataleoides*) and non-myrmecomorphic spiders (not identified to the species level but belonging to the same family, i.e., Salticidae) showed that the CVA1 axis clearly distinguished the mimics from non-mimics (Fig. 2a). Transformation grids, used to interpret shape changes associated with CVA1, demonstrated that the non-myrmecomorphic spiders (Non-mimics) were characterized by a broader cephalothorax and abdomen with a shorter pedicel than those of *Myrmarachne* spiders (Fig. 2b). The CVA2 axis revealed that the seven *Myrmarachne* species could be divided into two distinct groups: *M*. *maxillosa* and *M. malayana* had a positive score on the CVA2 axis (Group: Broad mimics), whereas the remaining five species had a negative score on the CVA2 axis (Group: Slender mimics). The body shape of the Slender mimics species was observed to be more slender and more elongated than that of the Broad mimics species, which had broader body forms. The discriminant function analysis (DFA) of the body shapes for pairwise comparisons among the Non-mimics group and the two groups of *Myrmarachne* species showed significant differences in body shape between the groups (Non-mimics vs. Broad mimics: the Mahalanobis distance = 9.51, T-square = 2204.17; Non-mimics vs. Slender mimics: the Mahalanobis distance = 7.89, T-square = 1968.28; Broad mimics vs. Slender mimics: the Mahalanobis distance = 6.73, T-square = 1048.02; Permutation tests of the Mahalanobis distances *P* < 0.001 for all comparisons; Fig. 3).

### Jumping distance comparisons

**Figure 4:**
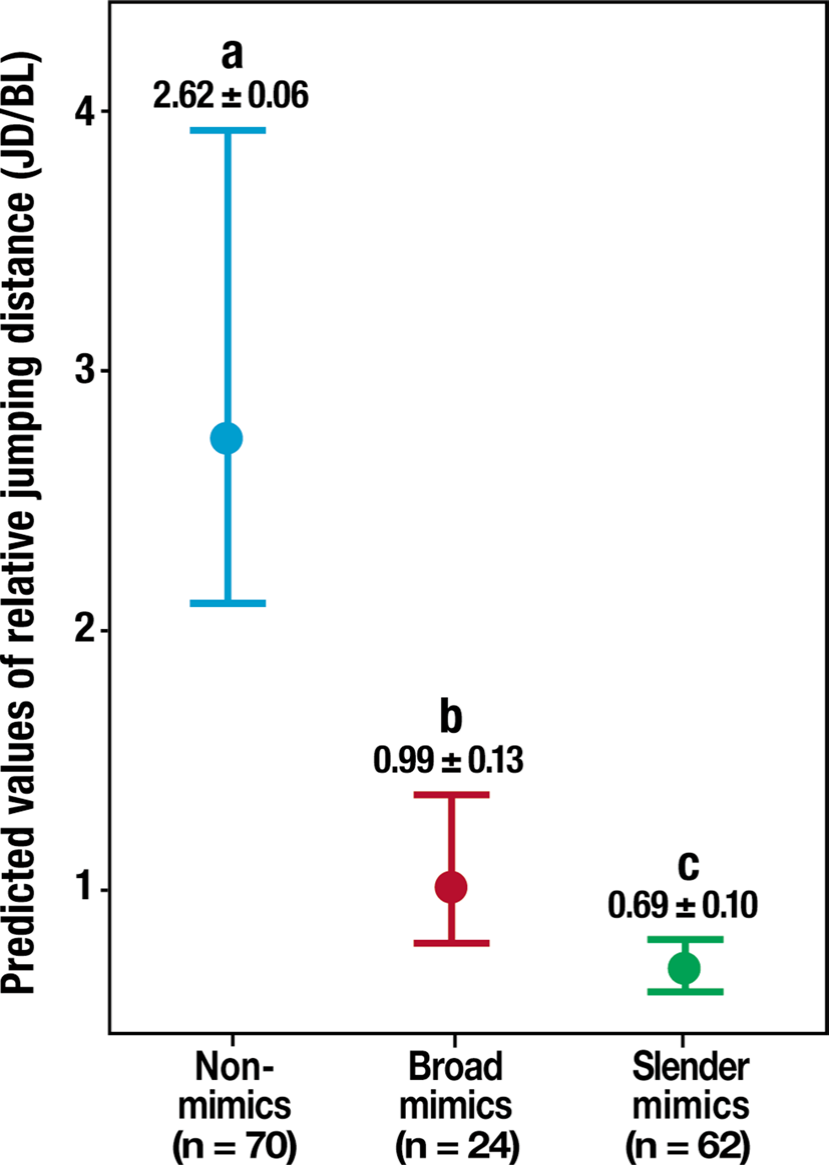
GLMM_g predicted probabilities of jumping distances for non-myrmecomorphic spiders (Non-mimics) and the two groups of *Myrmarachne* species (Broad mimics and Slender mimics). The vertical bands represent the 95% confidence intervals of the expected jumping distances. The values in the graph represent the mean ± standard error of the expected values. Different letters assigned to the boxes denote significant differences (Tukey’s HSD post hoc test *P* < 0.05). (Non-mimics: Non-myrmecomorphic spiders; Broad mimics: *M. maxillosa* and *M. malayana*; Slender mimics: *M. acromegalis*, *M. cornuta*, *M. hashimotoi*, *M. melanocephala*, and *M. plataleoides*).

**Figure 5:**
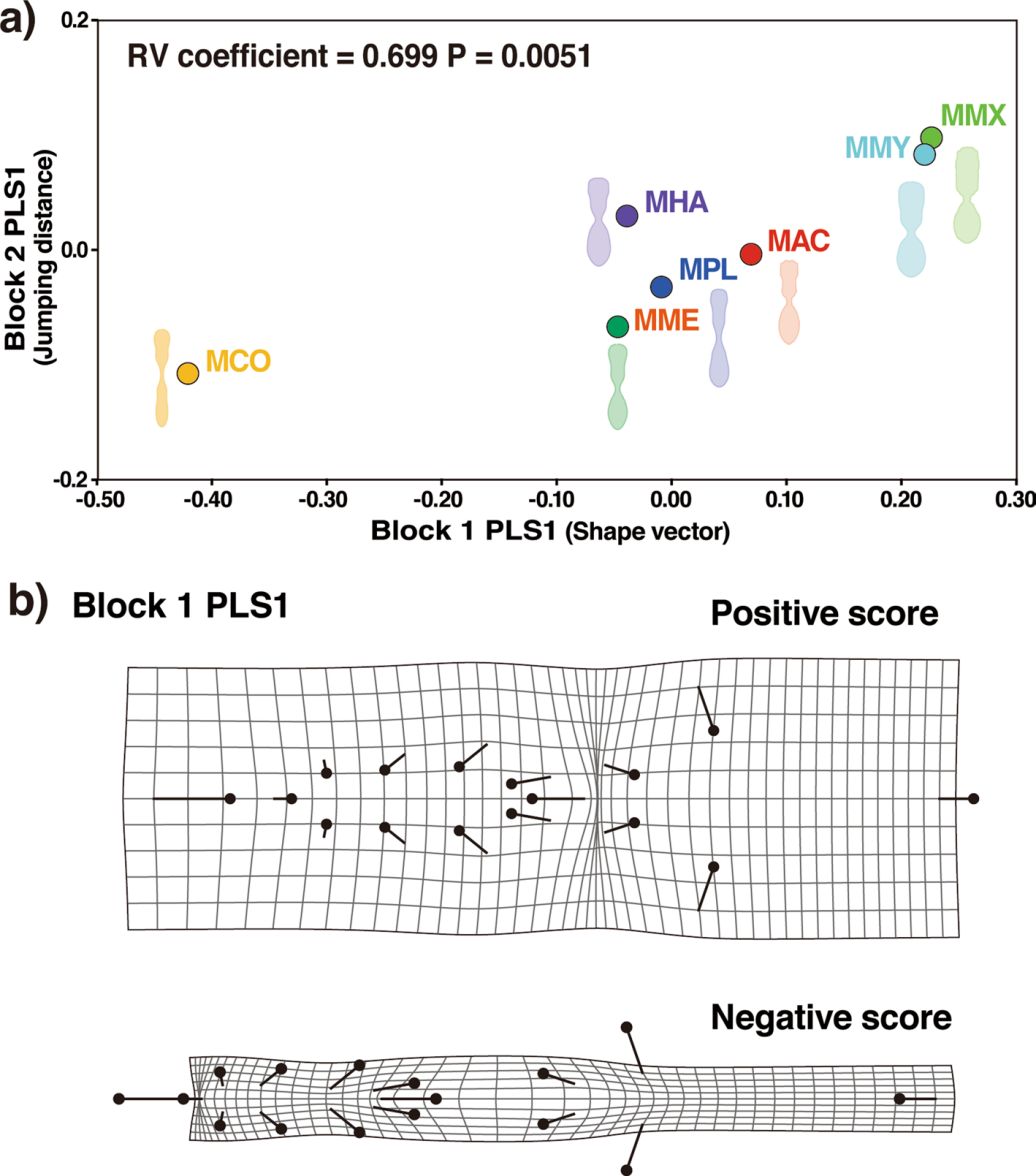
Relationship between the body shape and jumping distance of the seven *Myrmarachne* species. (**a**) Two-block partial least squares analysis (PLS) of the jumping distance against the mean body shape of the seven *Myrmarachne* species. The horizontal axis of the scatter plot represents the first PLS axis for body shape based on 16 landmarks and the vertical axis represents the first PLS axis for the jumping distance of the seven species. (MAC: *M. acromegalis*, MCO: *M. cornuta*, MHA: *M. hashimotoi*, MME: *M. melanocephala*, MMX: *M. maxillosa*, MMY: *M. malayana*, and MPL: *M. plataleoides*). (**b**) Transformation grids illustrate the shape changes associated with the PLS shape vector in positive and negative directions. (Box plots showing the variation in the jumping distances and wireframe outlines showing the mean body shapes of the seven *Myrmarachne* species have been provided in Supplementary Fig. S2).

Both groups of *Myrmarachne* species discriminated by CVA and DFA showed significantly shorter jumping distances than the Non-mimics, and the distances differed between the two groups (GLMM_g, χ^2^ = 102.63, df = 2, *P* < 0.001, Non-mimics: 2.81, CI = 2.50–3.12, Broad mimics: 1.00, CI = 0.80–1.21, Slender mimics: 0.68, CI = 0.57–0.79, the Tukey HSD test *P* < 0.05 for all comparisons; Fig. 4, Supplementary Table S1). The Slender mimics group presented shorter jumps than those exhibited by the Broad mimics group. The two-block partial least square (PLS) analysis revealed a high correlation between the body shape and jumping distance among the seven *Myrmarachne* species, indicating that the species with a shorter jumping distance typically had a slender body (RV coefficient = 0.699, *P* = 0.005; Fig. 5a, b).

### Prey-capture success rate comparisons

**Figure 6:**
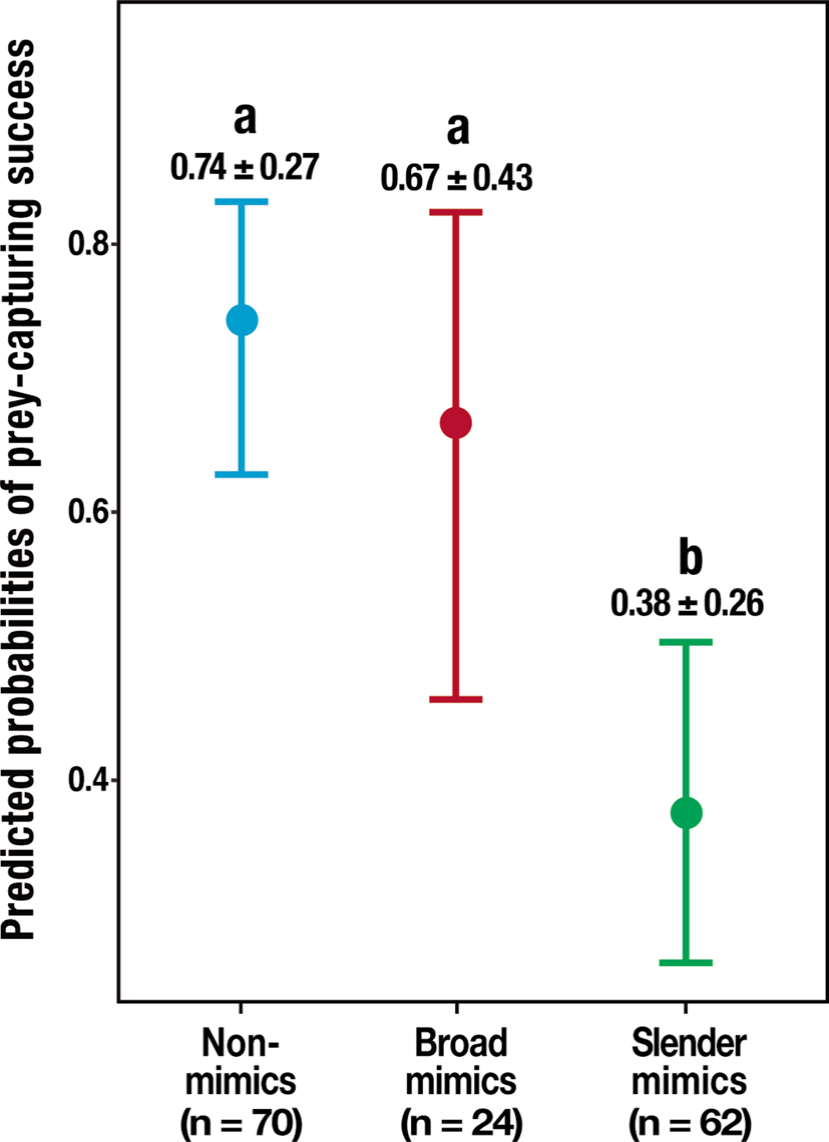
GLMM_b predicted probabilities of prey-capture success rates for non-myrmecomorphic spiders (Non-mimics) and the two groups of *Myrmarachne* species (Broad mimics and Slender mimics). The vertical bands represent the 95% confidence intervals of the expected prey-capture success rates. The values shown in the graph represent the mean ± standard error of the expected values. Different letters assigned to the boxes denote significant differences (Tukey’s HSD post hoc test *P* < 0.05). (Non-mimics: Non-myrmecomorphic spiders; Broad mimics: *M. maxillosa* and *M. malayana*; Slender mimics: *M. acromegalis, M. cornuta, M. hashimotoi, M. melanocephala*, and *M. plataleoides*).

Prey-capture success rate of *Myrmarachne* spiders were significantly lower than that of the Non-mimics (GLMM_b, χ^2^ = 17.77, df = 2, *P* < 0.001, Non-mimics: 0.74, CI = 0.63–0.83, Broad mimics: 0.67, CI = 0.46–0.82, Slender mimics: 0.38, CI = 0.27–0.50; Fig. 6, Supplementary Table S1). However, multiple comparisons showed no significant difference in the prey-capture rate between the Non-mimics and those in the Broad mimics group (Tukey’s HSD test *P* = 0.750). In contrast, the prey-capture success rate of the Slender mimics group was significantly lower than that of the Non-mimics group (Tukey’s HSD test *P* < 0.001).

## Discussion

This study provides, for the first time, quantitative evidence to support the hypothesis that the accurate morphological mimicry of ants by *Myrmarachne* jumping spiders may impose constraints on their jumping abilities and prey-capture success rates. Here, we employed geometric morphometric methods to quantify and discriminate the body shape variations in *Myrmarachne* and non-myrmecomorphic jumping spiders, and confirmed that *Myrmarachne* spiders had significantly slenderer and more elongated body shapes than non-mimics. The *Myrmarachne* spiders presented shorter jumping distances and lower prey-capture success rates than the non-myrmecomorphic jumping spiders, and the degree of these reductions was significantly correlated with the morphological changes resulting from the specific ant model selection. For example, among the *Myrmarachne* species investigated in the present study, *M. cornuta*, which mimics *Tetraponera* ants with an extremely slender body with elongated petiole, showed the shortest jumping distance to catch its prey. In contrast, *M. maxillosa* and *M. malayana*, which mimic *Polyrhachis* ants with more oval and stocky-shaped bodies, were able to jump to distances equal to their own body length to capture prey. Overall, these results suggested that reduction in jumping and prey-capture abilities of *Myrmarachne* spiders was associated with the morphological adaptations related to ant mimicry.

Given that *Myrmarachne* evolved from ancestor of a non-myrmecomorphic jumping spider^5^, the reduction in the jumping distance of *Myrmarachne* spiders may be explained by the unique jumping mechanism in spiders. The mechanism used by jumping spiders involves the use of a hydraulic catapult method in which spiders pump hemolymph fluid from the abdomen into the cephalothorax through the pedicel, and then increase the hemolymph pressure by compressing the cephalothorax^15,16^. In this hydraulic system, proper function requires an adequate enclosed space and fluid flow^16^. Therefore, the ant-like appearance of *Myrmarachne* spiders, such as narrow and constricted body with an elongated pedicel, seems unsuitable for generating the high hemolymph pressure required for jumping. Our geometric morphometric analysis, using a two-block PLS analysis, of *Myrmarachne* spiders further showed that the lower abilities of jumping were found in *Myrmarachne* species with narrower and more elongated forms. However, at present, no detailed studies have assessed the hydraulic mechanisms of jumping in *Myrmarachne* spiders. Thus, our assumption that the morphological ant mimicry by *Myrmarachne* spiders limits their hydraulic forces to jump should be addressed in future studies, for example, by analyzing the biomechanics of the hydraulic system in the mimetic spiders.

The present study showed that not only the jumping ability but also the prey-capture success rate was considerably reduced in *Myrmarachne* species with a slender body shape. As the results were only obtained under laboratory conditions, it is necessary to verify the prey-capture success rate under natural conditions via field observations. However, our results suggest the possibility that *Myrmarachne* species changed their diets in nature or may have evolved specialized prey-capture behavior to compensate for the poor jumping ability. Although none of the field evidence indicated that *Myrmarachne* species have a specialized predation behavior, such as snatching prey from ants as observed in *Menemerus* jumping spiders^20^, our previous study using stable isotope analysis demonstrated that *Myrmarachne* species with slender body shapes, such as *M. hashimotoi*, had low δ^15^N values similar to those of nectar-feeding ants and arthropod herbivores, but not similar to those of predators, in the same study sites^21^. In contrast, the δ^15^N values of *Myrmarachne* species with broad body shapes, such as *M. malayana* and *M. maxillosa*, were similar to those of omnivorous ants and non-myrmecomorphic jumping spiders. These findings indicated that plant-based diets were the main source of nutrition, especially for the species with slender body shapes, although some species of *Myrmarachne* have been observed to feed on the eggs of other spiders^22^. Jackson et al.^22^ and Nyffeler et al.^23^ reviewed previous studies on plant feeding by spiders and found that more than 40 species of jumping spiders, representing a dozen different subfamilies, were observed feeding on plant-derived food, such as nectar and honeydew, under natural conditions. Therefore, it is not surprising that some *Myrmarachne* spiders engage in phytophagy. However, plant-based diets are known to have a low protein content and are thus poor alternatives for animal prey^24^. If *Myrmarachne* spiders with slender body shapes indeed depend on plant-based diets as important food sources, this raises a further interesting question of whether the predominant plant feeding of *Myrmarachne* spiders interferes with their growth and fecundity. Clarification on the nutritional ecology of *Myrmarachne* spiders, with particular attention to their morphological resemblance to ants, will be a fascinating future research direction that will enable a better understanding of the costs of the mimetic resemblance.

In conclusion, our study showed that *Myrmarachn*e spiders were less efficient at capturing prey than non-myrmecomorphic jumping spiders, based on observations under laboratory conditions. Moreover, our geometric morphometric methods allowed us to quantitatively analyze the body shapes of *Myrmarachne* spiders, which revealed that the variations in their body shapes, resulting from specific model-mimic resemblances, were strongly associated with a reduction in their jumping and prey-capture abilities. In particular, a significant reductions in prey-capture proficiency was observed in *Myrmarachne* species with a slender and elongated body shape, which may influence these species to be phytophagous. Overall, the present study supported our hypothesis that the accurate morphological mimicry of ants by *Myrmarachne* spiders constrained the jumping and prey-capture abilities. Although our research focused on the constraints placed upon the jumping and prey-capture abilities due to mimicry, the accurate resemblance between *Myrmarachne* spiders and ants may potentially interfere with other activities because selection for mimicry can entail radical phenotypic changes in the mimics. Indeed, previous studies have suggested that the mimicry of *Myrmarachne* spiders imposes costs in the form of detriment effects on their life-history traits, such as mating^25,26^ and locomotion^27^. Furthermore, the resemblance between *Myrmarachne* spiders and ants can be achieved by not only body-form modifications but also combining a variety of morphological changes, all of which may potentially impose constraints on the mimics. For example, *Myrmarachne* spiders have thin legs like ants instead of the stout legs of typical salticids; however, thin legs were also found in non-myrmecomorphic salticids^28^. As salticid spiders usually use their legs to grasp and hold onto prey^29^, the thin legs of *Myrmarachne* spiders may also interfere with their prey-capture success rates. If the fitness costs of close mimetic resemblance greatly outweighs the benefits in a mimic species, then inaccurate mimicry may be selected for^10,30^. Therefore, to better understand how and why the accurate morphological mimicry evolved and is maintained in *Myrmarachne* spiders, we acknowledge that more studies should focus on the constraints imposed by the accurate morphological mimicry of ants. The methods used in the present study can be used in future investigations to provide novel insights into the evolutionary costs of accurate morphological mimicry in ant-mimetic jumping spiders.

## Methods

### Study site and study animals

The field sites and laboratories at each site were located at the Danum Valley Field Centre (4°55′N, 117°40′E) and the Lambir Hills National Park (4°20′N, 113°50′E) in Malaysian Borneo, and at the Sakaerat Environmental Research Station (14°30′N, 101°56′E) in northeastern Thailand. Sampling was conducted during four sampling events at the Danum Valley Field Centre-September 2004 (7 days), February 2005 (6 days), December 2006 (6 days), and January 2008 (5 days); five sampling events at the Lambir Hills National Park–August 2007 (7 days), July 2008 (7 days), February 2009 (9 days), September 2009 (5 days), and December 2012 (5 days); and three sampling events at the Sakaerat Environmental Research Station–March 2013 (6 days), September 2013 (5 days), and September 2014 (5 days). The spiders were collected on the day before testing for their jumping ability and prey-capture success rate and were held individually in plastic tubes containing a piece of damp cotton wool without food in the laboratory. For the tests, we used adult females and subadults of the spiders because they naturally spend much of their time foraging and not seeking partners. The subadults, which are the last instar before reaching sexual maturity, have almost the same size as that of the adults^31^. Small flies (1.5–3.0 mm body length) were used as prey items, which were collected in the field by sweep-netting on the day of testing. Eighty-six individuals from seven *Myrmarachne* species (MAC: *M. acromegalis*, n = 9; MCO: *M. cornuta,* n = 6; MHA: *M. hashimotoi*, n = 29; MMY: *M. malayana*, n = 14; MMX: *M. maxillosa*, n = 10; MME: *M. melanocephala*, n = 10; MPL: *M. plataleoides*, n = 8) were used in this study. Recently, Prószyński segregated 12 genera from the genus *Myrmarachne* without considering synapomorphies, or quantitative or phylogenetic analyses^32^. In addition, the monophyly of “*Myrmarachne*” in Prószyński’s sense is not supported, and his idea has not been followed by subsequent researchers^4,33^. Therefore, in this study we followed the previous definition of the genus *Myrmarachne*. Seventy individual non-myrmecomorphic jumping spiders were used in this study, including 12 salticid genera and unknown genera of the subfamily Salticinae (*Carrhotus*, n = 1; *Colyttus* , n = 1; *Cosmophasis*, n = 1; *Epeus*, n = 7; *Evarcha*, n = 1; *Parabathippus*, n = 5; *Phintella*, n = 2; *Ptocasius*, n = 23; *Rhene*, n = 3; *Simaetha*, n = 3; *Telamonia*, n = 3; *Thiania*, n = 3; and Unknown, n = 17) (pictures of the studied genera of non-myrmecomorphic salticids are shown in Supplementary Fig. S1).

### Geometric analysis

**Figure 7:**
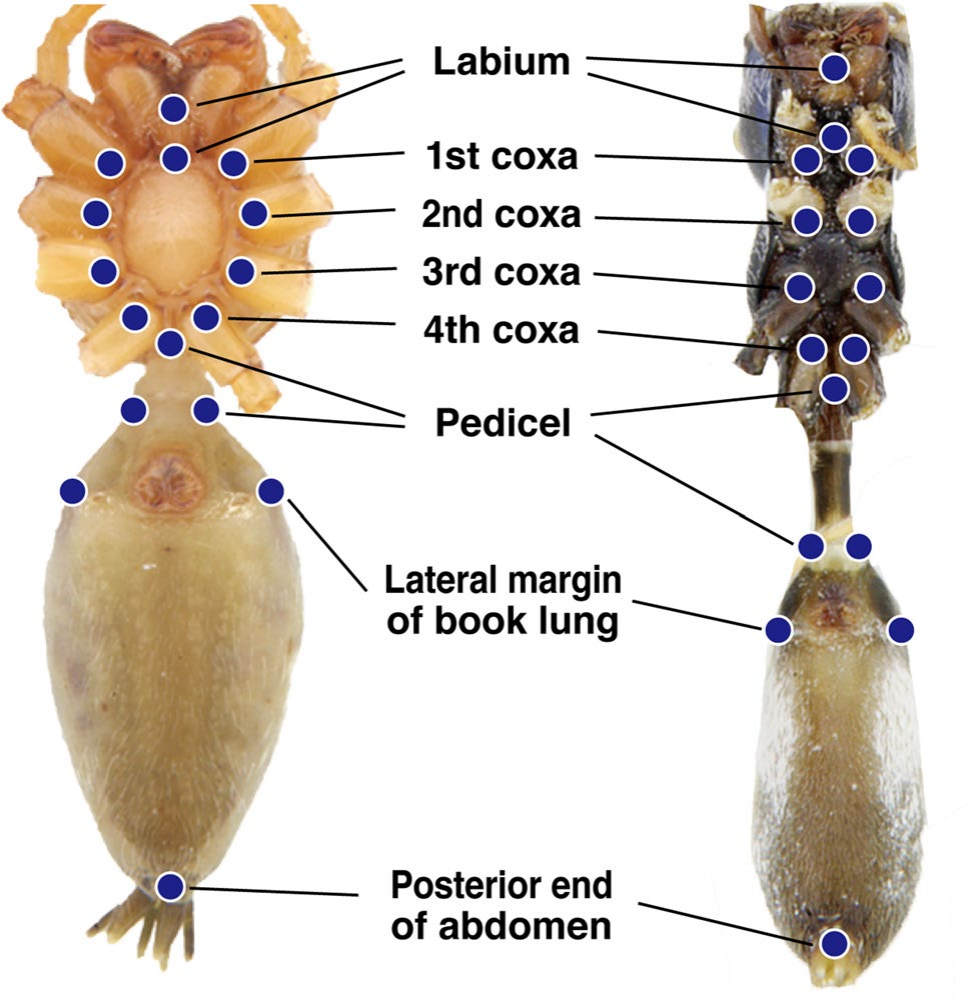
Ventral body views of non-myrmecomorphic (left) and *Myrmarachne* (right) jumping spiders showing the 16 landmarks used in this study.

Geometric analyses were performed using photographs of the ventral view of each spider body, where it was easy to determine the anatomically homologous points in both the non-myrmecomorphic and *Myrmarachne* jumping spiders. All photographs of the 86 individuals of the seven *Myrmarachne* species and the 70 individuals of non-myrmecomorphic salticids were taken using an Olympus digital camera (E-1) on a trinocular mount of a Leica MZ16a stereo-microscope. In each photograph, we digitized 16 landmarks using the ImageJ software version 1.50i (National Institute of Health, Bethesda, MD)^34^ (Fig. 7). The raw landmark coordinates were superimposed using a generalized procrustes analysis (GPA) to scale all configurations to the centroid size, translate them to the same location, and rotate them to the same orientation. Following the GPA, to visualize the body shape differences and group separation among spider taxa, we used CVA. DFA was used to test the statistical accuracy of the group separation indicated by CVA. For this analysis, the Mahalanobis distances, T-square tests, cross-validation scores, and wireframe results have been reported for pairwise comparisons among the groups. Permutation tests (10,000 rounds) with the T-square statistic for the Mahalanobis distance were performed to test the null hypothesis of equal group means. Cross-validation scores were used to assess the classification accuracy. Wireframe graphs of the mean and displaced landmarks described the most significant shape differences. Finally, a two-block PLS analysis was performed to assess the degree of association between the body shape and jumping distance. The two-block PLS analysis is a method to find a linear combination between two sets of data that can be used to analyze the relationship between shape and function^35^. The analysis provides an RV coefficient, which is analogous to a squared correlation coefficient and is a measure of the strength of association between two sets of variables. A permutation test with 10,000 permutations was performed to assess the statistical significance of this coefficient against the null hypothesis of complete independence between the two blocks of variables. All analyses were performed using MORPHOJ software (version 1.06)^36^.

### Testing procedures to measure the jumping distance and prey-capture success

All tests were conducted in a plastic cup (diameter: bottom, 9 cm and top, 10 cm, and height: 3.6 cm) (see Supplementary Fig. S3 and Supplementary Movie S1). The cup had a clear plastic top and a hole (diameter 1 cm) on the side. For each trial, a single spider and a single prey were introduced into the arena through the hole in the side of the cup. During the testing, the hole was covered with a cotton plug. The jumping distance and prey-capture success of the 86 individuals of the seven *Myrmarachne* species and the 70 individuals of non-myrmecomorphic salticids were recorded for 3 min with a video camera (Panasonic NV-GS202, VDR-D300, and Sony HDR-CX590V) that was positioned above the arena. Jumping distance was measured from screen captures using Adobe Photoshop CC (Adobe Systems Inc) and the distance at which the spider launched its attack was measured between the head of the spider and the prey. The longest jump by each spider was selected and used in the data analysis. If spiders only performed one jump, this distance was used in the analysis. The relative jumping distance was used to compare the jumping distance of spiders, whereby the jumping distance (JD) was divided by the body length (BL). Each spider was used just once per test. The spiders that did not respond to the prey in their trials were not included in the analysis.

### Statistical analysis

To compare the jumping distances and prey-capture successes between non-myrmecomorphic jumping spiders and the *Myrmarachne* species, we used generalized linear mixed models (GLMMs). Owing to the small sample size of each *Myrmarachne* species, we aggregated the seven species into groups that were discriminated by CVA and DFA, and included “species of *Myrmarachne* and genus of non-myrmecomorphic salticids” as a random intercept effect in the GLMMs to control for potential differences among species within each group. We used a GLMM with gamma distribution (GLMM-g) to compare the jumping distances, and GLMM with binomial distribution and logistic regression link (GLMM-b) to compare the prey-capture success. Analysis of the GLMMs was performed using the R package lme4 (version 1.1–21)^37^. A post hoc pairwise comparison of our GLMM estimated means was conducted using Tukey’s HSD test and the R package multcomp (version 1.4–10)^38^. All statistical analyses were performed using R (version 3.60)^39^.

## Supplementary information

Supplementary Video 1.

Supplementary Information 1.

Supplementary Information 2.
