# Supplementary material for: Constraints on the jumping and prey-capture abilities of ant-mimicking spiders (Salticidae, Salticinae, Myrmarachne)
Source: Sci Rep. 2020 Oct 26;10:18279. doi: 10.1038/s41598-020-75010-y (PMC7589502; doi:10.1038/s41598-020-75010-y)
Supplement: Supplementary file 3 — Supplementary Information 2. [file 41598_2020_75010_MOESM3_ESM.pdf]

Supplementary Information Files for the Paper:

Constraints on the jumping and prey-capture abilities of ant-mimicking spiders (Salticidae, Salticinae, Myrmarachne)

Authors: Yoshiaki Hashimoto[1*], Tomoji Endo[2], Takeshi Yamasaki[1], Fujio Hyodo[3], Takao Itioka[4]

[1]University of Hyogo/ Museum of Nature and Human Activities, 6 Yayoigaoka, Sanda, Hyogo 669-1546, Japan.

[2]Shool of Human Sciences, Kobe College, Okadayama 4-1, Nishinomiya, Hyogo, 662-8505, Japan.

[3]Research Core for Interdisciplinary Sciences, Okayama University, 3-1-1, Tsushimanaka, Okayama, 700-8530, Japan.

[4]Graduate School of Human and Environmental Studies, Kyoto University, Yoshida-nihonmatsu, Kyoto 606-8501, Japan.

*Corresponding author: Yoshiaki Hashimoto [E-mail: yoshiaki@hitohaku.jp]

Journal: Scientific Reports

Supplementary Dataset S1: Data of the relative jumping distance (JD/BL) , prey-capture success and body length of the ant-mimicking and non-mimicking spiders. Attached Excel file contains 2 data sheets for 1) Relative jumping distance. 2) Prey-capture success.

Supplementary Movie S1: A collection of videos including the testing arena and prey-capture behaviors of *Ptocasius* sp. (non-mimicking salticid) and *Myrmarachne hashimotoi* (ant-mimicking salticid) .

Supplementary Figures S1, S2, S3 and Table S1: Three additional supportive figures and table; please see below.

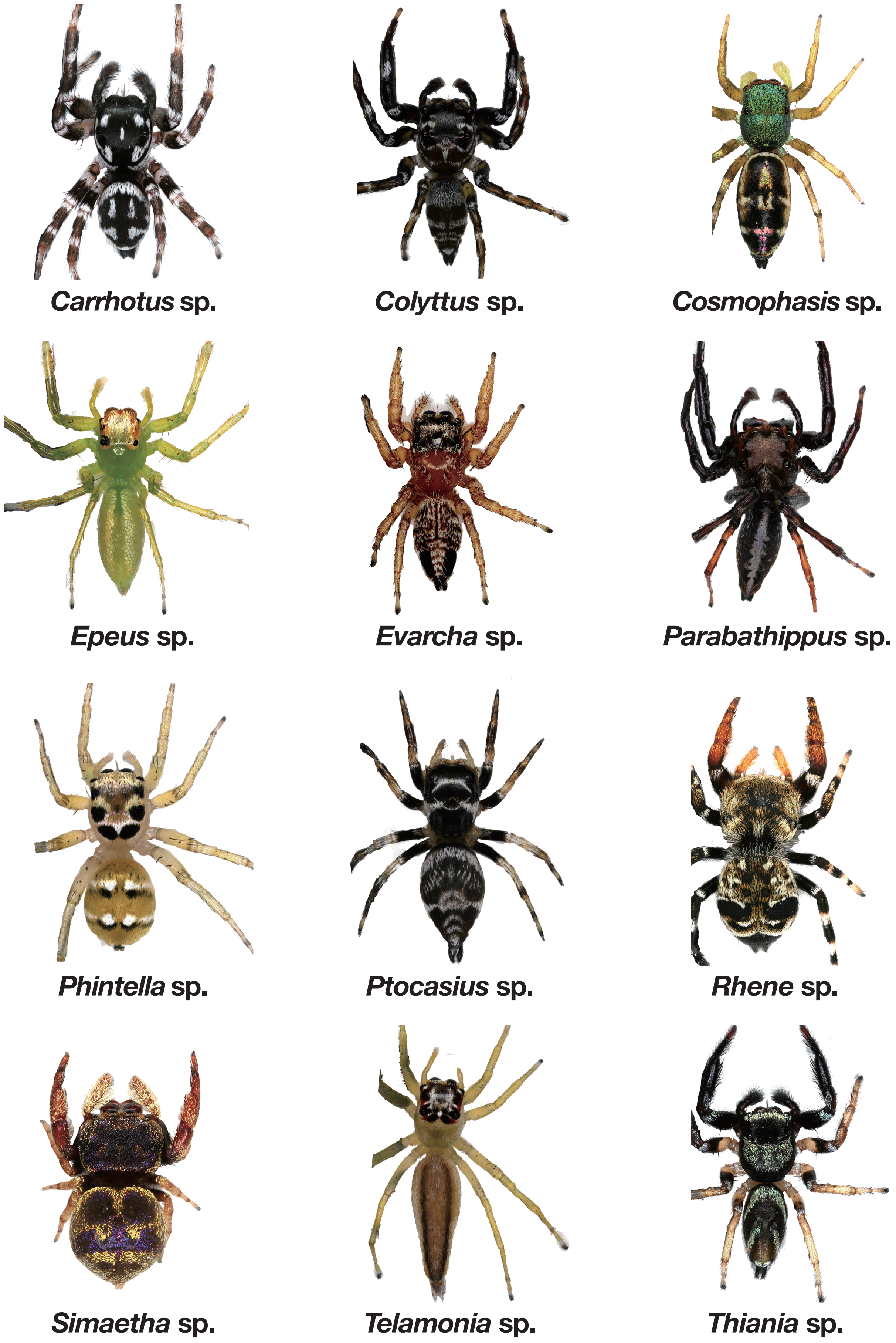

Fig. S1. Studied genera of non-mimetic salticids, except unknown genera.

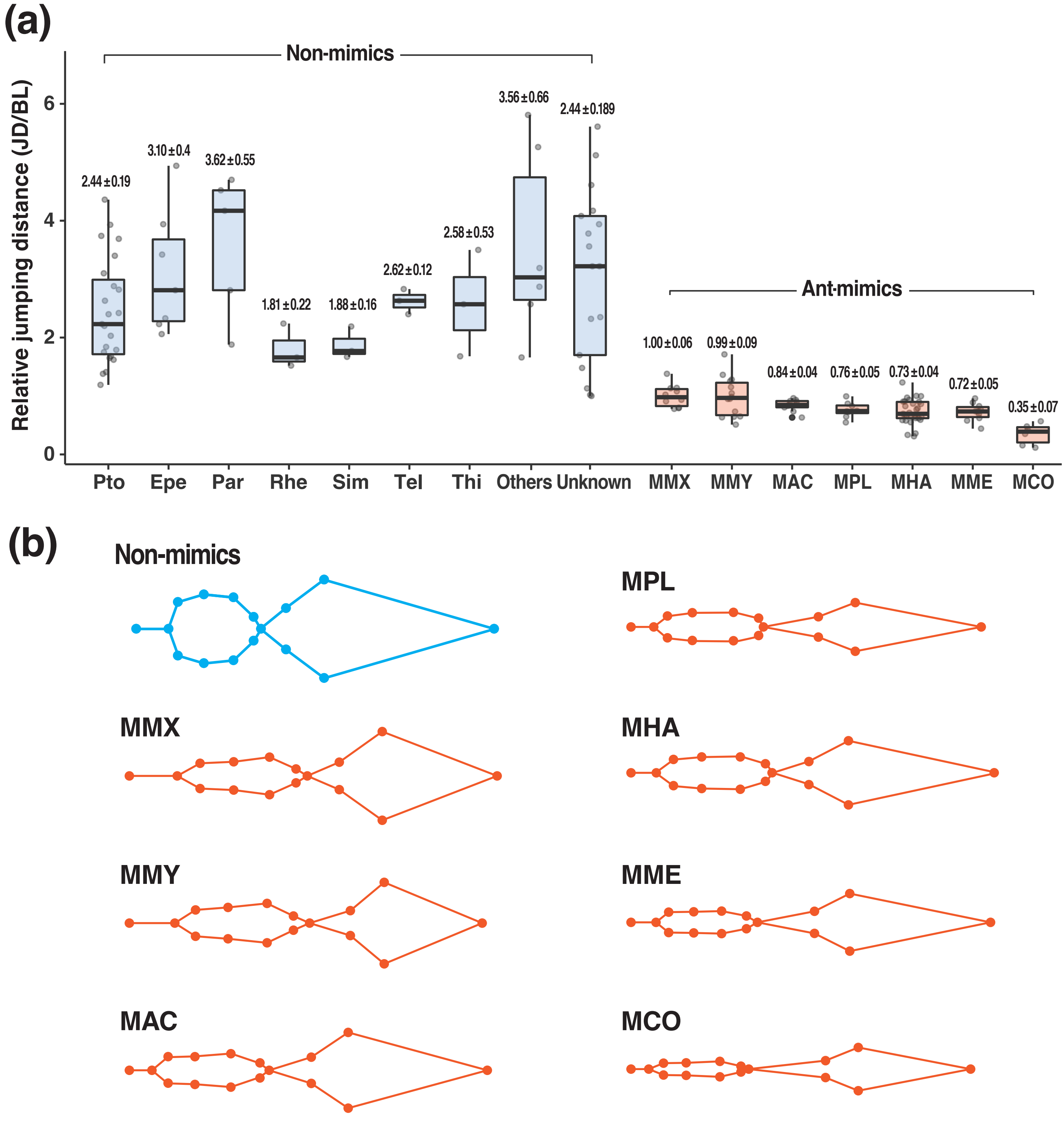

Fig. S2. (**a**) Box plot showing variation in relative jumping distances (jumping distance/body length) of Non-mimetic salticids and seven *Myrmarachne* species. Boxes extend from the 25th to 75th percentiles, with the band indicating median; whiskers represent the 5th and 95th percentiles. Values shown above boxes are the mean ± standard error. (**b**) Mean body-shape of Non-mimetic salticids and each *Myrmarachne* species are demonstrated with wireframe outlines. (Plto: *Ptocasius*, Epe: *Epeus*, Par: *Parabathippus*, Rhe: *Rhene*, Sim: *Simaetha*, Tel: *Telamonia*, Thi: *Thiania*, MAC: *M. acromegalis*, MCO: *M. cornuta*, MHA: *M. hashimotoi*, MME: *M. melanocephala*, MMX: *M. maxillosa*, MMY: *M. malayana*, MPL: *M. plataleoides*).

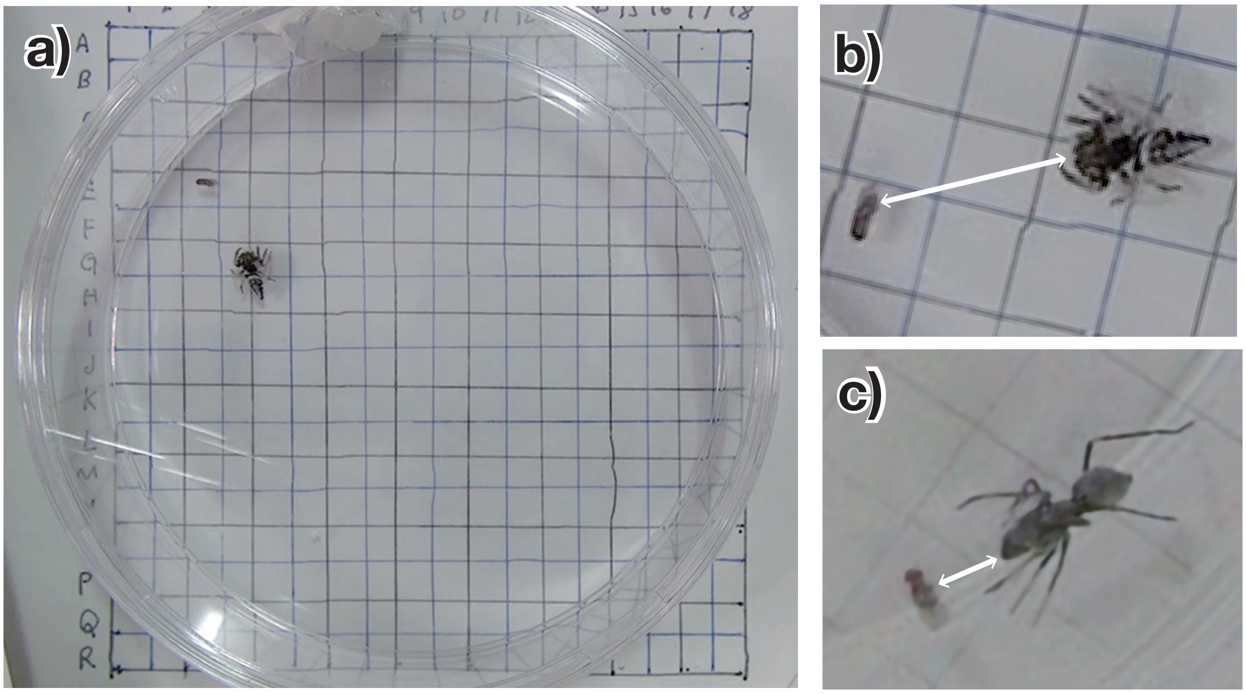

Fig. S3. Testing apparatus used to record jumping distance and prey-capture success of spiders. **(a)** Testing arena. The arena is comprised of a clear plastic cup (9 cm diameter on bottom, 10 cm diameter on top, and 3.6 cm height) with a clear plastic top and a hole (diameter 1 cm) in the side to introduce the prey. First, we dropped a spider into the arena and then put a fly (1.5-3 mm body length) through the hole in the side of the cup. During the experiment, the hole was covered with a cotton plug. **(b)** and **(c)** Measurement of jumping distance. Salticids stalk their prey and stop to prepare for jumping at the prey from a certain distance. The distance from prey position to the stopping position of spiders is measured as the jumping distance. Spiders that did not respond to the prey in their trials were not included in the analysis. (**b**: *Ptocasius* sp., **c**: *Myrmarachne maxillosa*)

Table S1. Summary and predicted probabilities of the GLMMs comparing jumping distances (a) and prey-capture successes (b) among Non-mimics, Broad mimics and Slender mimics.

(a) GLMM_g<-glmer(JD~Group+(1|Species & genus), data=da, family = Gamma(link = "inverse"))

	**Jumping distances**			
Predictors	*Estimates*	*CI*		*p*
(Intercept)	1.46	1.31	1.64	**<0.001**
MYR-B	1.88	1.42	2.50	**<0.001**
MYR-S	2.90	2.34	3.60	**<0.001**
**Random Effects**				
σ^2	0.11			
$\tau_{00\ Sp}$	0.01			
ICC	0.08			
N_{Sp}	20			
Observations	156			
Marginal R^2 / Conditional R^2	0.669 / 0.694			

	**Jumping distances**			
	Predicted	*SE*	*CI*	
Non-mimics	2.62	0.06	3.73	2.02
Broad mimics	0.99	0.13	1.32	0.78
Slender mimics	0.69	0.10	0.80	0.61

(b) GLMM_b<-glmer(c(S+F)~Group+(1|Species & genus), data=da, family = binomial("logit"))

	**Prey-capturing successes**			
Predictors	*Odds Ratios*	*CI*		*p*
(Intercept)	2.89	1.69	4.94	**<0.001**
MYR-B	0.69	0.25	1.89	0.473
MYR-S	0.21	0.10	0.44	**<0.001**
**Random Effects**				
σ^2	3.29			
$\tau_{00\ Sp}$	0.00			
N_{Sp}	20			
Observations	20			
Marginal R^2 / Conditional R^2	0.124 / NA			

	**Prey-capturing successes**			
	Predicted	*SE*	*CI*	
Non-mimics	0.74	0.27	0.63	0.83
Broad mimics	0.67	0.43	0.46	0.82
Slender mimics	0.38	0.26	0.27	0.50
